# Prognostic Risk Factors of 30-Day Death in Traumatic Lower Limb Fracture Patients with Acute Pulmonary Embolism: A Single-Center Retrospective Study

**DOI:** 10.1155/2023/8246730

**Published:** 2023-05-10

**Authors:** Huanhuan Li, Yijun Yu, Yuting Wang, Qian Zhang, Ye Gu

**Affiliations:** ^1^Department of Cardiology, Wuhan Fourth Hospital, Wuhan, Hubei, China; ^2^Department of Ultrasonic Imaging, Wuhan Fourth Hospital, Wuhan, Hubei, China

## Abstract

**Background:**

To explore the prognostic risk factors of 30-day death in patients with traumatic lower limb fracture (TLLF) complicated with acute pulmonary embolism (APE).

**Methods:**

295 consecutive TLLF patients diagnosed as APE according to pulmonary artery CT angiography, hospitalized in our hospital from January 2017 to December 2021, were included in this study. Patients were divided into nonsurvival group and survival group according to 30-day follow-up results. After adjusting age, sex, and all the clinical variables with *P* values of <0.2 with backward stepwise method (likelihood ratio), multivariate Cox regression analysis was used to analyze risk factors of 30 days all-cause death in TLLF patients with APE. The area under curve (AUC) calculated by receiver operating characteristic curve (ROC) and the incremental model were used to determine the prognostic potential of identified risk factors.

**Results:**

29 patients died during 30-day follow-up. Simplified pulmonary embolism severity index (sPESI) score ≥1 (*P* < 0.05), Wells score ≥7 (*P* < 0.01), and pulmonary hypertension (*P* < 0.01) were associated with higher risk, while anticoagulant therapy (*P* < 0.01) was associated with lower risk of all-cause death during 30 days follow-up in APE patients. Compared with sPESI score, Wells score plus pulmonary hypertension produced better predictive efficacy. Prognostic value of sPESI score could be enhanced by adding Wells score, pulmonary hypertension, and anticoagulant therapy to the predicting models.

**Conclusions:**

Wells score ≥7 and pulmonary hypertension are independent predictive risk factors of 30-day all-cause death in TLLF patients with APE.

## 1. Introduction

Acute pulmonary embolism (APE) is an urgent clinical disease, which is the third leading cause of cardiovascular death after acute myocardial infarction and stroke [1, 2]. High mortality and increased morbidity of APE were observed worldwide over the past decade [3, 4]. Relevant study showed that the hospital based incidence rate of APE was 0.1% per year in China from 1997 to 2008 [5]. Awareness of prognostic risk factors of short-term all-cause death post APE is of importance to reduce mortality in patients with APE, since APE-related death ranged from a few hours to days after onset, with more than 70% mortality occurring within the first hour after APE [6, 7].

Patients with traumatic lower limb fracture (TLLF) faced a higher risk of deep venous thrombosis (DVT) and APE due to severe trauma, orthopedic surgery, and perioperative bed rest or immobilization [8]. The purpose of this study was to determine the predictive risk factors of 30-day death in TLLF patients with APE.

## 2. Materials and Methods

### 2.1. Study Population

**Figure 1:**
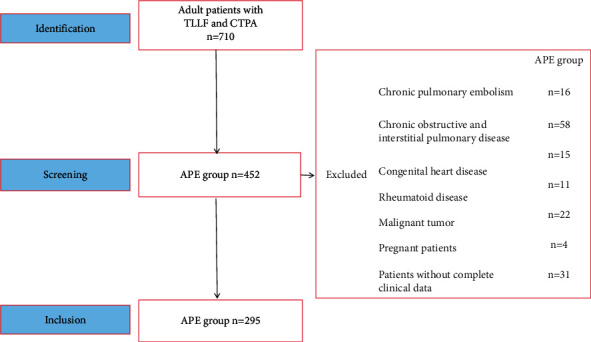
Diagram of patient flow.

Strobe statement [9] was used for patients screening, identification, and inclusion (shown in Figure 1). In this retrospective study, 710 consecutive patients with TLLF, who were admitted in our hospital from January 2017 to December 2021 and underwent pulmonary artery computed tomography angiography (CTPA) examination for suspected APE were selected. Patients with signs of dyspnea, chest pain, threatened syncope, syncope, and/or hemoptysis were defined as suspected APE patients [3]. All patients were scored according to Wells scoring system, including clinical signs and symptoms of DVT, likelihood of alternate diagnosis is unlikely heart rate >100/min, immobilization or surgery in the previous 4 weeks, prior DVT/PE, hemoptysis, and malignancy (treatment currently, in the previous 6 months, or palliative) [10]. Patients with chronic pulmonary embolism, chronic obstructive and interstitial pulmonary disease, congenital heart disease, rheumatoid disease, malignant tumor, and pregnant were excluded. Patients without complete clinical data were also excluded. Finally, 295 TLLF patients with APE were included in this study. According to 30-day followup results, patients were divided into nonsurvival group (*n* = 29) and survival group (*n* = 266). Simplified pulmonary embolism severity index (sPESI) clinical scores were calculated for all patients diagnosed with APE [11]. All survived patients at follow-up gave informed consent for participation in this study and informed consent was obtained from the next of kin (*s*)/legal guardian (*s*) for all patients who died during follow-up. The study protocol was approved by the ethical committee of our hospital (approval number: KY-2020-131-01) and conducted in accordance with the ethical principles of the Declaration of Helsinki.

### 2.2. Diagnostic Criteria of APE

**Figure 2:**
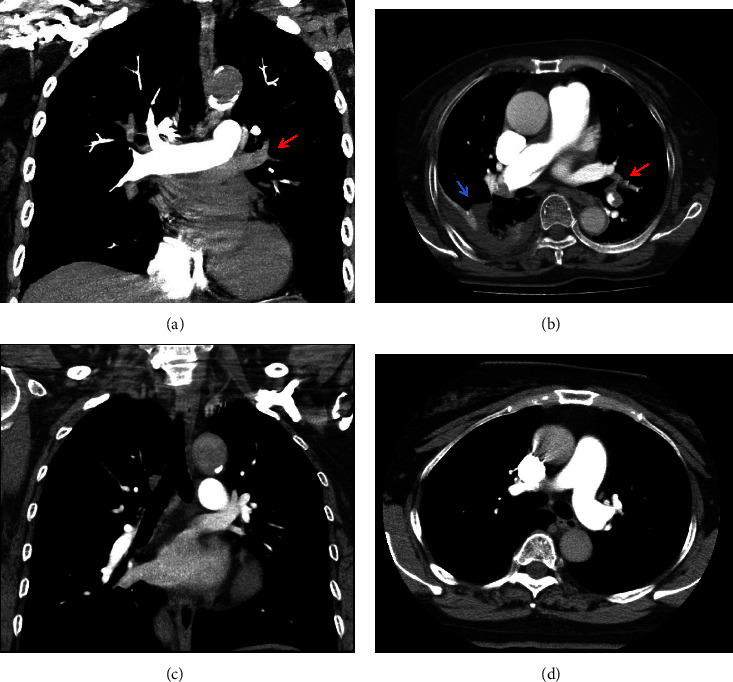
CTPA images in the APE and non-APE patients. (a) Complete filling defect in left main pulmonary artery filling without development of distal vessels (red arrow) in chest longitudinal section of soft-tissue window in APE patients. (b) Filling defect of the distal left pulmonary artery (red arrow) and atelectasis of the right lower lung with pleural effusion (blue arrow) in chest cross section of soft-tissue window in APE patients. (c) There was no filling defect in bilateral pulmonary arteries in chest longitudinal section of soft-tissue window in non-APE patients. (d) There was no filling defect in bilateral pulmonary arteries in chest cross section of soft-tissue window in non-APE patients.

TLLF patients with suspected APE received CTPA examination after admission. The diagnosis of APE was based on CTPA results according to the 2019 European Society of Cardiology (ESC) guidelines [3] for the diagnosis and treatment of APE. The direct signs of APE were low-density filling defect in pulmonary artery, partial or complete “track sign” surrounded by opaque blood flow or complete filling defect without development of distal vessels [12] (shown in Figure 2(a)). The indirect signs were wedge-shaped high-density area or discoid atelectasis in the lung field, dilated of central pulmonary artery, and reduced or disappeared of distal vessels [12] (shown in Figure 2(b)). There was no filling defect in bilateral pulmonary arteries in chest longitudinal section of soft-tissue window (shown in Figure 2(c)) and chest cross section of soft-tissue window (shown in Figure 2(d)) in non-APE group.

### 2.3. CTPA Examination

**Figure 3:**
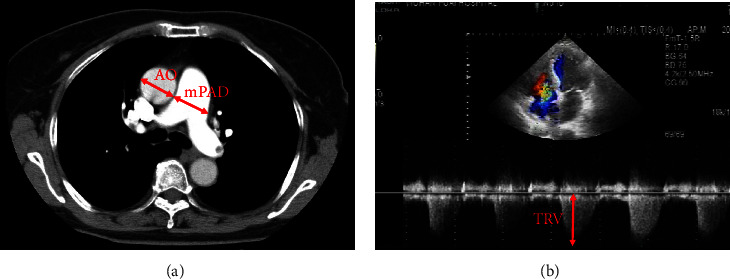
Pulmonary hypertension images by CTPA and echocardiography. (a) mPAD and AO in CTPA (red arrow). (b) TRV was measured (red arrow).

CTPA was performed with plain lung scan and pulmonary artery enhanced scan by Toshiba aquilion CXL64 slice spiral CT (Toshiba company, Japan). The CTPA images were analyzed as previously described [13] with Vitrea Software (Toshiba company, Japan). The widest diameters of the main pulmonary artery (mPAD) and ascending aorta (AO) on the horizontal section of the bifurcation were measured using an electronic caliper within 3 cm from the bifurcation of the main pulmonary artery (shown in Figure 3(a)). The diameter ratio of diameter of the main pulmonary artery to the ascending aorta (rPA) was calculated.

### 2.4. Echocardiography Examination

Echocardiography examination was performed according to the guidelines of the American Society of Echocardiography (ASE) [14]. Tricuspid regurgitation velocity (TRV) was measured (shown in Figure 3(b)). The tricuspid regurgitation gradient (TRG) was calculated from the TRV (=4 × TRV^2^) [15].

### 2.5. Pulmonary Hypertension

Pulmonary hypertension was defined as mPAD ≥ 29 mm and rPA ≥1 measured by CTPA [13] or TRG ≥ 31 mmHg by echocardiography [16].

### 2.6. Primary Endpoint

All patients were followed up for 30 days by clinical visit or telephone interview. The primary endpoint was all-cause death.

### 2.7. Statistical Analysis

Kolmogorov–Smirnov test was used for normal distribution of all continuous variables. Continuous variables with normal distribution were expressed as mean ± standard deviation and compared by two-tailed Student's *t*-test. Categorical variables were expressed as count and percentages and compared by Chi-square test. Survival analysis was compared by log rank test. Cox proportional hazards models were used to identify factors that predict adverse outcomes (i.e., all-cause death). The hazard ratios (HRs) for the 95% confidence intervals (CIs) were calculated. Age, sex, and all the clinical variables with *P* values of <0.2 in univariable regression were included in the multivariable regression model, and independent factors were determined using a backward stepwise method (likelihood ratio). The diagnostic potential of risk factors for all-cause death during 30 days follow-up were analyzed by receiver operating characteristic curve (ROC), and the performance of different prediction models was judged on the basis of the c-statistic (area under the receiver operating characteristic curves). Incremental model performance was assessed by changes in the Chi-square value for the regression models with enter method. Statistical analyses were performed with SPSS17.0 software (IBM Company, USA). *P* < 0.05 was statistically significant.

## 3. Results

### 3.1. Follow-Up Results and Clinical Characteristics

29 APE patients died during the 30-day follow-up, due to heart failure (*n* = 14), severe pulmonary infection (*n* = 11) and acute myocardial infarction (*n* = 4).  Table 1 showed the clinical characteristics between the nonsurvival group and the survival group in TLLF patients with APE. Age ≥65, sPESI score ≥1, Wells score ≥7, and pulmonary hypertension were significantly higher in the nonsurvival group than in the survival group (all *P* < 0.05). Incidence of DVT was similar between the two groups. There was no inferior vena cava (IVC) thrombosis in this patient cohort.

9 patients did not receive anticoagulant therapy in the nonsurvival group due to following reasons: active gastrointestinal bleeding (*n* = 2), active bleeding at fracture site (*n* = 4), and traumatic intracranial hemorrhage (*n* = 3). 26 patients did not receive anticoagulant therapy in the survival group due to following reasons: active gastrointestinal bleeding (*n* = 5), active bleeding at fracture site (*n* = 12), and traumatic intracranial hemorrhage (*n* = 9). Percent of anticoagulant therapy was significantly lower in the nonsurvival group than in the survival group (*P* < 0.01). Among the patients with anticoagulant therapy contraindications, the number of patients treated with IVC filter in the survival group was significantly more than that in the nonsurvival group (*P* < 0.05). No patients underwent surgical embolectomy. There was no significant difference in antiplatelet therapy between the two groups.

### 3.2. Predictors of All-Cause Mortality in TLLF Patients with APE

**Figure 4:**
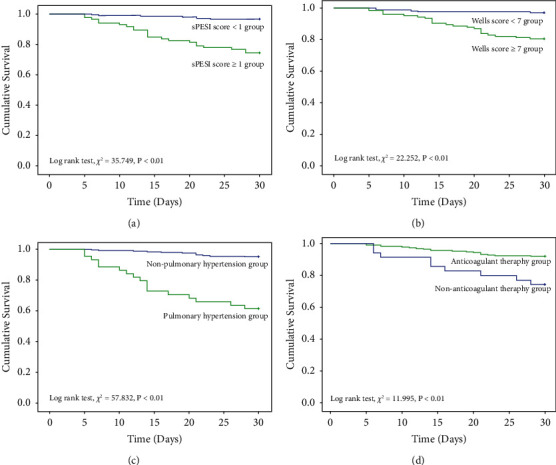
Kaplan–Meier curve of 30-day survival rate in patients with TLLF complicated with APE. (a) sPESI score. (b) Wells score. (c) Pulmonary hypertension. (d) Anticoagulant therapy.

During 30-day follow-up, in TLLF patients with APE, Kaplan–Meier curves demonstrated that the risk of all-cause death were significantly higher in the sPESI score ≥1 group than in the sPESI score <1 group (log rank test, *χ*^2^ = 35.749, *P* < 0.01, Figure 4(a)), in the Wells score ≥7 group than in the Wells score <7 group (log rank test, *χ*^2^ = 22.252, *P* < 0.01, Figure 4(b)), in the pulmonary hypertension group than in the nonpulmonary hypertension group (log rank test, *χ*^2^ = 57.832, *P* < 0.01, Figure 4(c)), and the risk of all-cause death were significantly lower in the anticoagulant therapy group than in the nonanticoagulant therapy group (log rank test, *χ*^2^ = 11.995, *P* < 0.01, Figure 4(d)).

As shown in the Table 2, univariable predictors of all-cause mortality included age ≥65, sPESI score ≥1, Wells score ≥7, and pulmonary hypertension. Multivariate Cox regression analysis showed that sPESI score ≥1 (HR = 3.370, 95% CI 1.265–8.978, *P* < 0.05), Wells score ≥7 (HR = 6.243, 95% CI 2.357–16.541, *P* < 0.01), and pulmonary hypertension (HR = 5.169, 95% CI 2.234–11.958, *P* < 0.01) were independently predictors of increased all-cause mortality. Anticoagulant therapy (HR = 0.343, 95% CI 0.151–0.781, *P* < 0.01) was an independently predictor of lower all-cause mortality (Table 2).

### 3.3. Diagnostic Performance of Wells Score and Pulmonary Hypertension on the Prognosis of APE Patients

**Figure 5:**
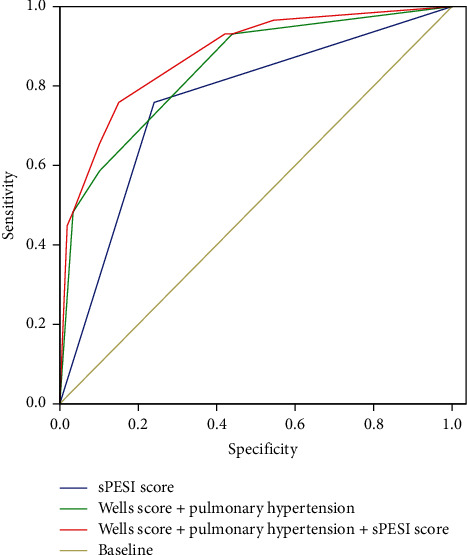
ROC analysis and AUC calculation.

ROC curve analysis was performed to evaluate the prognostic diagnostic potential of sPESI score, Wells score plus pulmonary hypertension for all-cause death of APE (Figure 5). The performance statistics of the prediction model showed that only sPESI score showed good prediction utility (*c* statistics: 0.759). Deformation predictors Wells score plus pulmonary hypertension produced better predictive efficacy (*c* statistic: 0.842). Adding deformation predictors Wells score and pulmonary hypertension to sPESI score produced very good predictive efficacy (*c* statistic: 0.877). Other approaches yielded no further favorable utility, such as Wells score (*c* statistic 0.728) and pulmonary hypertension (*c* statistic 0.742) (Table 3).

Incremental predictive efficacy on 30-day all-cause death of the PESI score ≥1, Wells score ≥7, pulmonary hypertension, and anticoagulant therapy in APE patients.

**Figure 6:**
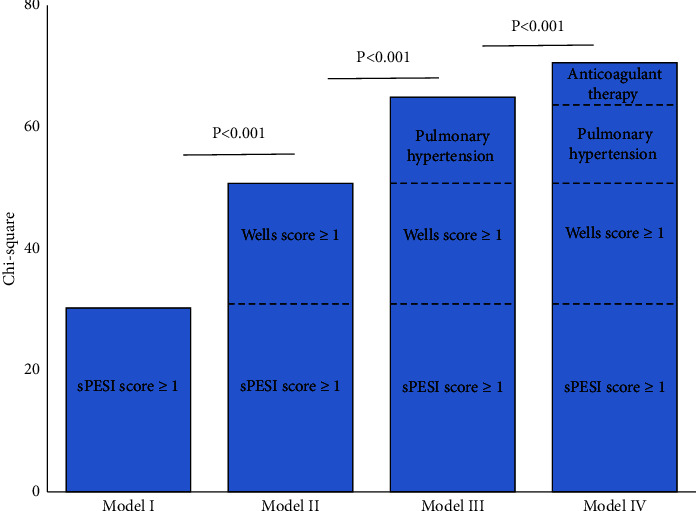
Incremental model performance for predicting prognosis assessed by starting with the clinical variables (model I PESI score ≥1), followed by Wells score ≥7 (model II: adding Wells score ≥7 to model I), then followed by pulmonary hypertension (model III: adding pulmonary hypertension to model II), and finally by adding anticoagulant therapy (model IV).

The sPESI score ≥1 was entered in the first step of a multivariable Cox model to predict all-cause death (Chi-square 30.265, *P* < 0.001). In Model II, adding Wells score ≥7 to the Model I enhanced the explanatory power (Chi-square 50.700, *P* < 0.001 vs. Model I). Adding pulmonary hypertension (Model III) further improved the prognostic performance of the Model II (Chi-square 64.898, *P* < 0.001 vs. Model II). Adding anticoagulant therapy (Model IV) further improved the prognostic performance of the Model III (Chi-square 70.588, *P* < 0.001 vs. Model III) (Figure 6).

## 4. Discussion

The major findings of the present study were as follows: (1) sPESI score ≥1, Wells score ≥7, and pulmonary hypertension were independent predictors of 30-day all-cause death, while anticoagulant therapy was an independent protector of 30-day all-cause death for TLLF patients complicated with APE. (2) Deformation predictor and Wells score plus pulmonary hypertension had better prognostic diagnostic potential on outcome than sPESI score in TLLF patients complicated with APE. (3) Prognostic value of sPESI score could be enhanced by adding Wells score, pulmonary hypertension, and anticoagulant therapy to the predicting models. The results of this study suggested that TLLF patients with confirmed APE, the Wells score ≥7, pulmonary hypertension, and no anticoagulant therapy are independent prognostic determinants of worse outcome, and these patients should be closely monitored and treated to reduce the risk of mortality within 30 days post APE.

APE is the general term of a group of diseases and clinical syndromes, which refer to various embolisms that block the pulmonary artery or its branch system. In China, the incidence of APE is increasing year by year, the mortality is high, and the long-term prognosis is poor. An outpatient study showed that the 90-day all-cause mortality rate of APE was as high as 11.1% [17]. Traumatic fracture refers to the interruption of bone integrity or continuity caused by accident or violence [18, 19]. TLLF patients are known to prone to APE because they have the three elements of Virchow for venous thrombosis (hypercoagulant state, vascular endothelial injury, and venous congestion) [20]. CTPA is the first-line examination method for the diagnosis of APE [12, 21]. CTPA examination was used to diagnosis APE in this patient cohort.

Due to the complexity of clinical diagnosis of APE, we used Wells scoring system [10, 22, 23] recommended by ESC guidelines [3] to evaluate TLLF patients with suspected APE. We used the original version of the three classification methods for evaluation the possibility of APE, with Wells score ≥7 indicating high risk probability, and Wells score <7 representing low and medium risk probability [10]. The ESC guidelines [3] emphasized the importance of RV function assessment of APE, because it could identify high-risk patients before they had clinical deterioration. Studies showed that in individuals without cardiopulmonary diseases, when APE occurred, 25–30% of the pulmonary vessels were blocked and the right ventricle produced an acute mean pulmonary arterial pressure of up to 40 mmHg, the pulmonary arterial pressure would rise [24, 25]. Therefore, pulmonary hypertension was selected to evaluate the risk and prognosis in the patients with TLLF combined with APE. In our study, both echocardiography [16] and CTPA [26–28] were used to define pulmonary hypertension.

After the diagnosis of APE, we conducted risk assessment for patients according to ESC guidelines [3]. We selected the widely used sPESI score, which had been proved to be a predictor of 30 days all-cause death in patients with APE as benchmark [11, 29, 30]. The result showed that sPESI score ≥1 (HR = 3.370, 95% CI 1.265–8.978, *P* < 0.05), Wells score ≥7 (HR = 6.243, 95% CI 2.357–16.541, *P* < 0.01), and pulmonary hypertension (HR = 5.169, 95% CI 2.234–11.958, *P* < 0.01) were independently predictors of increased all-cause death of TLLF complicated with APE. Meanwhile, ROC curve analysis showed that compared with sPESI score, the combination of Wells score, and pulmonary hypertension could improve the prediction effect of 30-day all-cause death in patients with TLLF and APE. Incremental model showed that adding Wells score and pulmonary hypertension to sPESI score could enhance the prognostic value. Compared with multiple data collection of sPESI score, Wells score had been evaluated when patients suspected of APE, and the method of obtaining pulmonary hypertension data is easier and more objective. Our results are the first to define the 30-day death prognostic value of Wells score and pulmonary hypertension in TLLF patients with APE.

The ESC guidelines [3] recommend anticoagulant therapy after the diagnosis of APE to prevent early death and recurrence of VTE. We found that after APE was diagnosed, anticoagulant therapy (HR = 0.343, 95% CI 0.151–0.781, *P* < 0.01) was independently predictor of decreased all-cause death of APE in TLLF patients. Our study suggested an increased risk of all-cause death in TLLF patients who did not receive anticoagulant therapy after the diagnosis of APE. 59 patients who received nonpermanent IVC filter (temporary filter or recyclable filter) implantation due to contraindication of anticoagulant therapy or large, free floating proximal DVT, and underwent bone surgery operation after filter implantation. If there were no contraindications to anticoagulant therapy, removed the nonpermanent IVC filters after DVT was stable. When patients had anticoagulant therapy contraindications, our results showed that the number of patients treated with IVC filter in the survival group was significantly more than that in the nonsurvival group (*P* < 0.05). Our study showed that when anticoagulant contraindications exist in TLLF patients with APE, IVC filter should be implanted according to ESC guidelines [3]. At the same time, patients should be dynamically evaluated for persistent anticoagulant contraindications. Removal of the IVC filter should be considered once anticoagulant therapy is resumed without recurrent bleeding complications.

Our study showed that sPESI score, Wells score, and pulmonary hypertension were independent predictors of increased 30-day all-cause death, while anticoagulant therapy was an independent predictor of lower all-cause death in TLLF patients complicated with APE. Combined assessment with Wells score and pulmonary hypertension enhanced the predicting efficacy on 30-day death outcome in TLLF patients complicated with APE.

### 4.1. Study Limitations

This study is a retrospective study based on a single-center database. Multicenter database with larger patient cohort is essential to validate present results.

## 5. Conclusions

Prevalence of APE is high among TLLF patients. Wells score ≥7 and pulmonary hypertension are independent prognostic risk factors for 30-day all-cause death of TLLF patients complicating APE.

## Figures and Tables

**Table 1:** Clinical characteristic of nonsurvival group and survival group in APE patients.

	Nonsurvival group (*n* = 29)	Survival group (*n* = 266)	*P* value
Male (*n*, %)	15 (51.72)	121 (45.49)	0.328
Age (yr) ≥65	23 (79.31)	159 (59.77)	0.029
BMI (kg/m^2^)	22.23 ± 1.67	22.07 ± 1.66	0.617
Smoking history (*n*, %)	8 (27.59)	74 (27.82)	0.586
Drinking history (*n*, %)	6 (20.69)	50 (18.80)	0.484
Hypertension (*n*, %)	14 (48.28)	110 (41.36)	0.300
Type 2 diabetes (*n*, %)	5 (17.24)	39 (14.66)	0.441
Hyperlipidemia (*n*, %)	9 (31.03)	64 (24.06)	0.268
Hyperuricemia (*n*, %)	5 (17.24)	31 (11.65)	0.269
Cerebral infarction (*n*, %)	6 (20.69)	44 (16.54)	0.365
Length of stay (days)	15.17 ± 7.02	16.64 ± 6.12	0.227
Bone surgery operation (*n*, %)	26 (89.66)	249 (93.61)	0.312
DVT (*n*, %)	20 (68.97)	204 (76.69)	0.238
sPESI score ≥1 (*n*, %)	21 (72.41)	69 (25.94)	<0.01
Wells score ≥7 (*n*, %)	24 (82.76)	99 (37.22)	<0.01
Pulmonary hypertension (*n*, %)	17 (58.62)	27 (10.15)	<0.01
Anticoagulant therapy after APE diagnosis	20 (68.97)	240 (90.23)	0.003
Low molecular weight heparin (*n*, %)	16 (55.17)	168 (63.16)	
Vitamin K antagonists (*n*, %)	1 (3.45)	9 (3.38)	
Thrombin inhibitors (*n*, %)	1 (3.45)	24 (9.02)	
Coagulation factor Xa inhibitor (*n*, %)	2 (6.90)	39 (14.66)	
Systemic thrombolysis (*n*, %)	2 (6.89)	10 (3.76)	0.334
Percutaneous catheter directed treatment (*n*, %)	4 (13.79)	31 (11.65)	0.460
IVC filter implantation (*n*, %)	8 (27.59)	59 (22.18)	
In nonanticoagulant therapy patients (*n*, %)	4 (44.44)	22 (84.62)	0.030
In anticoagulant therapy patients (*n*, %)	4 (20.00)	37 (15.42)	0.390
Antiplatelet therapy after APE diagnosis (*n*, %)	3 (10.34)	15 (5.64)	0.255

APE, acute pulmonary embolism; BMI, body mass index; DVT, deep venous thrombosis; sPESI: simplified pulmonary embolism severity index; IVC: inferior vena cava.

**Table 2:** Predictors of all-cause death in TLLF patients complicated with APE by Cox proportional hazards regression.

	Unadjusted HR (95% CI) *P* value				Adjusted^*∗*^ HR (95% CI) *P* value			
Age ≥65	2.466	1.004	6.456	0.049	1.022	0.988	1.057	0.204
Sex	1.264	0.610	2.618	0.529	1.940	0.920	4.092	0.082
sPESI score ≥1	8.600	3.672	20.141	<0.01	3.370	1.265	8.978	0.015
Wells score ≥7	7.220	2.754	18.928	<0.01	6.234	2.357	16.541	<0.01
Pulmonary hypertension	10.150	4.840	21.286	<0.01	5.169	2.234	11.958	<0.01
Anticoagulant therapy	0.274	0.125	0.602	0.001	0.343	0.151	0.781	0.011

HR: hazard ratio; other abbreviations as in Table 1. ^*∗*^Adjustment for age ≥65, sex, and all the clinical variables with *P* values of <0.20 with backward stepwise method (likelihood ratio).

**Table 3:** Model performance statistics for predicting 30-day all-cause mortality in patients with TLLF and APE.

	Discrimination (ROC, c-statistic)			
	*c*-statistic	95% CI		*P* value
sPESI score	0.759	0.664	0.854	<0.001
Wells score	0.728	0.639	0.817	<0.001
Pulmonary hypertension	0.742	0.63	10.854	<0.001
Wells score + pulmonary hypertension	0.842	0.764	0.920	<0.001
Wells score + pulmonary hypertension + sPESI score	0.877	0.809	0.946	<0.001

Abbreviations as in Tables 1 and 2.

## Data Availability

All data generated or analyzed during this study are included in this article. Further inquiries can be directed to the corresponding author.

## Abbreviations

ACCP: American College of Chest Physicians
AO: Ascending aorta
APE: Acute pulmonary embolism
BMI: Body mass index
CI: Confidence interval
CTPA: Pulmonary artery computed tomography angiography
DVT: Deep venous thrombosis
ESC: European Society of Cardiology
HR: Hazard ratio
IVC: Inferior vena cava
mPAD: Main pulmonary artery diameter
OR: Odds ratio
sPESI: Simplified pulmonary embolism severity index
PTE: Pulmonary thromboembolism
rPA: The ratio of main pulmonary artery to ascending aorta
SPAP: Pulmonary artery systolic pressure
TLLF: Traumatic lower limb fracture
TRV: Tricuspid regurgitation velocity
V/Q: Ventilation/blood flow ratio
VTE: Venous thromboembolism.
